# 890. Financial Hardship After Burn Injury: Feasibility and Early Patient-reported Findings from a Longitudinal Cohort Study

**DOI:** 10.1093/jbcr/irag033.376

**Published:** 2026-04-06

**Authors:** Maiya I Pacleb, Caitlin M Orton, Carly Marincasiu, Joseph Grobowski, Simrat Kaur, Robyn Wilkinson, John W Scott, Barclay T Stewart

**Affiliations:** University of Washington School of Medicine, Seattle; University of Washington Medicine Regional Burn Center, Harborview Medical Center, Seattle, WA; Department of Surgery, University of Washington Medicine Regional Burn Center, Harborview Medical Center, Seattle, WA; Department of Surgery, University of Washington Medicine Regional Burn Center, Harborview Medical Center, Seattle, WA; University of Washington Medicine Regional Burn Center, Harborview Medical Center, Seattle, WA; University of Washington Medicine Regional Burn Center, Harborview Medical Center, Seattle, WA; University of Washington, Seattle, WA; University of Washington, Seattle, WA; Department of Surgery, University of Washington School of Medicine / Harborview Injury Prevention and Research Center, Seattle, WA

## Abstract

**Introduction:**

Financial hardship, the objective financial burden and subjective distress related to medical care, affects 1 in 4 working-age trauma survivors. Like trauma, burn injuries involve care costs and lost work that strain health, function, and recovery. Income survey measures are poorly collected among burn-injured adults, with nearly 1 in 5 patients declining to respond. When reported, income alone fails to reveal the true burden of financial hardship, ignoring out-of-pocket costs, non-medical expenses, and financial worry. We aimed to evaluate the feasibility of including financial hardship survey items into a longitudinal study and provide an early description of financial hardship among adults with burn injuries.

**Methods:**

Adult participants enrolled in a longitudinal cohort study were asked to complete surveys that included a new 5-item financial hardship measure developed by the American Association for the Surgery of Trauma, with adapted pre-injury questions and Spanish translation. Surveys were collected at discharge (pre-injury recall) or at 6, 12, or 24 months post-injury. Descriptive statistics summarized demographics and financial hardship survey responses and completion rates were assessed to evaluate feasibility.

**Results:**

Fifty-seven participants were offered the financial hardship survey, with 95% completing all survey items (Fig. 1). Income was reported by 74%. At discharge, 20 of the 21 participants completed all items, and 39 of 40 participants surveyed at follow-up completed all items. Medical bill concerns were reported by 14% of pre-injury and 22% of post-injury respondents. Non-medical bill concerns were reported by 19% of pre-injury and 45% of post-injury respondents. Delays in care due to costs were reported by 29% of pre-injury and 15% of post-injury respondents. Financial worry was reported for 76% of pre-injury and 60% of post-injury respondents.

**Conclusions:**

Financial hardship surveys were feasible to collect, with high participant response rate. This pilot was not powered to detect pre- and post-injury differences but reported responses provide early insights. Consistent with other populations (e.g., cancer, trauma), post-injury respondents reported problems with medical and non-medical bills, and financial worry remained highly prevalent. In contrast, delays in care were less frequent, potentially mitigated by specific resources in the burn center (e.g., integrated multidisciplinary care, vocational rehabilitation counseling, support for low income and unhoused participants). These findings underscore survey feasibility for those living with burn injury and the need for longitudinal research to clarify the prevalence of financial hardship in burn-injured populations.

**Applicability of Research to Practice:**

This study demonstrates the feasibility of financial hardship surveys in burn research, supporting future longitudinal studies to examine their prevalence and guide mitigating interventions.

**Funding for the study:**

The contents of this abstract were developed under a grant from the National Institute on Disability, Independent Living, and Rehabilitation Research (NIDILRR grant #90DPBU0005). NIDILRR is a Center within the Administration for Community Living (ACL), Department of Health and Human Services (HHS). The contents of this abstract do not necessarily represent the policy of NIDILRR, ACL, HHS, and do not assume endorsement by the Federal Government.

